# Hypothesis on the functional advantages of the selection-broadcast cycle structure: global workspace theory and dealing with a real-time world

**DOI:** 10.3389/frobt.2025.1607190

**Published:** 2025-11-13

**Authors:** Junya Nakanishi, Jun Baba, Yuichiro Yoshikawa, Hiroko Kamide, Hiroshi Ishiguro

**Affiliations:** 1 Graduate School of Engineering Science, The University of Osaka, Osaka, Japan; 2 AI Lab, CyberAgent Inc., Tokyo, Japan; 3 Faculty/School of Law, Kyoto University, Kyoto, Japan

**Keywords:** global workspace theory, selection, broadcast, serial processing, parallel processing, real-time world

## Abstract

This paper discusses the functional advantages of the Selection-Broadcast Cycle structure proposed by Global Workspace Theory (GWT), inspired by human consciousness, particularly focusing on its applicability to artificial intelligence and robotics in dynamic, real-time scenarios. While previous studies often examined the Selection and Broadcast processes independently, this research emphasizes their combined cyclic structure and the resulting benefits for real-time cognitive systems. Specifically, the paper identifies three primary benefits: Dynamic Thinking Adaptation, Experience-Based Adaptation, and Immediate Real-Time Adaptation. This work highlights GWT’s potential as a cognitive architecture suitable for sophisticated decision-making and adaptive performance in unsupervised, dynamic environments. It suggests new directions for the development and implementation of robust, general-purpose AI and robotics systems capable of managing complex, real-world tasks.

## 1 Introduction

In recent years, a major research theme in the fields of artificial intelligence (AI), robotics, and cognitive science has been how to implement the advanced intelligence and flexible problem-solving abilities of humans and animals into systems Hassabis et al. (2017); Ho and Griffiths (2022). For example, large language models (LLMs) learn from vast amounts of text data through attention mechanisms and acquire the ability to respond flexibly to unknown questions Vaswani et al. (2017). This capability is seen as going beyond mere pattern matching and mimicking some of the human cognitive functions such as reasoning and knowledge integration. Similarly, in the field of image recognition, deep learning-based feature extraction techniques have made significant progress, leading to practical applications in various tasks such as object detection, face recognition, and scene understanding Edozie et al. (2025); Ni et al. (2023). While the development of learning and recognition technologies in individual modalities is remarkable, the advanced intelligence exhibited by humans and animals is characterized by their ability to adapt to the environment by integrating multiple sensory modalities rather than relying on a single information source. As a result, there is growing interest in “multimodal” and “parallel” architectures that execute tasks by simultaneously utilizing multiple cognitive functions Kotseruba and Tsotsos (2020); Ajay et al. (2023). However, the integration of information and complementary reasoning between these specialized modules (e.g., visual, linguistic, logical reasoning, and motor control) remain limited, and this is one of the major challenges in the field Liu et al. (2025).

Against this background, the Global Workspace Theory (GWT), which was devised by imitating human consciousness, is attracting attention. GWT positions “consciousness” from the perspective of information processing structure and proposes a framework in which information that has been competed for and integrated among numerous parallel specialized modules is temporarily brought “into consciousness” and then shared system-wide Baars (2005). Since it was first proposed by the psychologist Bernard Baars, GWT has been linked to many empirical findings in neuroscience and cognitive science Dehaene and Naccache (2001); Mashour et al. (2020). More recently, its advantages as an information processing architecture have begun to attract attention in AI research as well. Previous GWT research suggests that the “Selection” process, which integrates information among multiple parallel specialized modules, and the “Broadcast” process, which disseminates the selected information throughout the system, are expected to be effective as a wide range of functions, including creative thinking, transfer learning, top-down control, and attention allocation Mashour et al. (2020); Juliani et al. (2022); VanRullen and Kanai (2021).

However, there is one perspective that continues to be overlooked in many of these discussions. That is information processing with a temporal dimension, i.e., not a single process or static environment, but a chain of multiple information processing operations that require careful consideration to find an answer, and responses to dynamically changing environments. These information processing methods with a temporal dimension are important research topics in artificial intelligence systems that handle complex tasks that require learning and adaptation, and in robotics, where real-time processing is required Lesort et al. (2020); Shaheen et al. (2022); Shiwa et al. (2008); Alhaddad et al. (2020). For example, in unknown complex tasks, it is not possible to arrive at an answer with a single processing step, and it is necessary to consider various perspectives and organize information to derive an answer. Furthermore, in tasks performed in dynamic environments, sensor data is updated moment by moment, and task goals and external conditions change depending on the situation. In short, the essence of intelligence lies not only in single-shot input processing, but also in the flow of information processing that unfolds as a temporal chain. However, in traditional GWT research has been limited to mentioning the usefulness of “Selection” and “Broadcast” separately, i.e., in a static, single process, and the effectiveness of executing these two processes in parallel and intermittently (i.e., information processing with a temporal dimension) has not been sufficiently addressed.

This study focuses on the process of information exchange through selection and broadcast, hereinafter refers to this as the “Selection-Broadcast Cycle”, and aims to fill this gap. In other words, we position the Selection-Broadcast Cycle as an extension of conventional GWT that introduces a temporal dimension, thereby reinforcing the theoretical standing of GWT as an information processing architecture for specialized module integration. To this end, based on conceptual processing flow, we have presented a hypothesis regarding the functional advantages provided by its dynamic and cyclical structure. Specifically, we propose the following three hypotheses.

Dynamic Thinking Adaptation: a capacity to dynamically rearrange module execution order, thereby enabling flexible adaptation to unexpected task changes or evolving goals Experience-Based Adaptation: an acceleration of consciousness processing by exploiting past experiences stored in memory modules, facilitating faster predictions and decision-making Immediate Real-Time Adaptation: a quick intervention route to consciousness processing allows for immediate response to real-time changes.

The contributions of this study can be summarized in the following two points. First, this paper introduces a temporal dimension that was lacking in previous GWT research and formalizes a new structure called the Selection-Broadcast Cycle. In previous frameworks, selection and broadcast were primarily treated as discrete events, but this study redefines them as dynamic and cyclical processes, providing a theoretical foundation that explains sequential information updates and environmental adaptation. Second, through conceptual processing flow, this study verified the functional advantages of the Selection-Broadcast Cycle in real-time adaptability and long-term task execution. This suggests that the proposed framework is not merely a theoretical concept but has potential utility in practical applications, including robotics and dialogue agents.

## 2 Literature review

### 2.1 Overview of GWT

**FIGURE 1:**
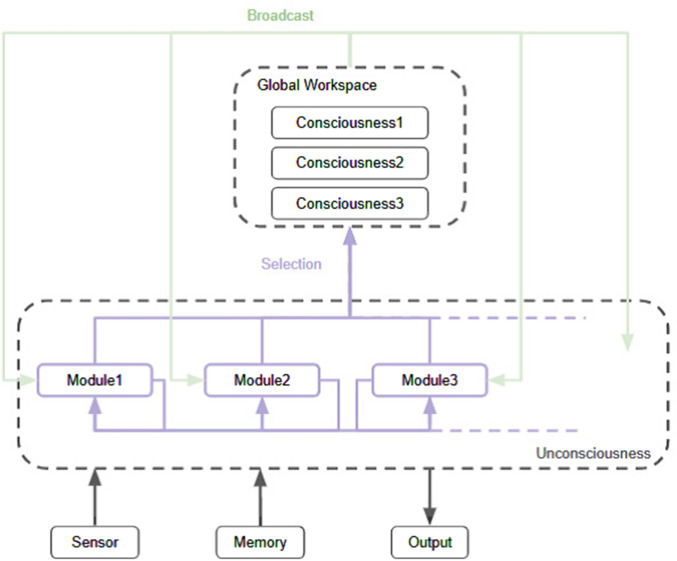
Architecture of the global workspace theory.

The Global Workspace Theory (GWT) is a cognitive science theory of information processing in consciousness, proposed by the psychologist Bernard Baars (2005). The essence of GWT is a framework in which information is competed and integrated among many specialized modules (e.g., vision, hearing, memory, language) that operate in parallel, and the information that eventually wins is then shared among all modules (Figure 1). The winning information is temporarily retained in a conscious form within a memory area called the “global workspace”. Only a limited amount of information can win at a time, and other competing information is considered to be processed unconsciously in the background. In this way, GWT is positioned as a framework to explain the interaction between a serial, limited-capacity conscious process and parallel, large-capacity unconscious processes. This model is supported by numerous experimental findings Dehaene and Naccache (2001); Mashour et al. (2020). For example, in brain imaging studies (e.g., fMRI, PET, EEG), stimuli processed under consciousness involve extensive regions of the brain, including the frontal and parietal lobes. These stimuli exhibit recurrent signaling, whereas stimuli that do not reach conscious awareness (i.e., go through unconscious processing) remain confined to local, transient activity Dehaene et al. (2001); Gaillard et al. (2009). This is consistent with the mechanism proposed by GWT that once some piece of information wins, it is broadcast globally to the entire system.

These demonstrate one aspect of conscious information processing, but many mysteries remain regarding practical implementation. For example, the formalization of selection criteria for which information is selected and shared globally remains insufficient. It has been suggested that the integrated weighting of bottom-up attention (e.g., the intensity of sensory stimuli) and top-down control (e.g., current task goals) may be a determining factor in selection Buschman and Miller (2007), but the specific computational mechanisms by which this is evaluated remain unclear. Additionally, the conditions under which these weightings dynamically change and the underlying control principles remain unexplored. For example, reward prediction errors or reinforcement learning feedback may adjust the criteria for what information is considered important. However, how such learning processes are integrated within the GWT framework remains unclear both theoretically and empirically. Furthermore, it is necessary to clarify what information is actually exchanged in the global workspace. In the current model, the unit of information is ambiguous, with sensory representations, semantic symbols, and behavioral intentions coexisting. Which granularity of information is suitable for global propagation, and how it is formatted (e.g., from sensory to linguistic) requires further experimental and computational investigation. At the same time, the diversity of information processing units that may exist as specialized modules and their constitutive conditions remain ambiguous. In GWT, the parallelism of modules is discussed abstractly, but the mapping to actual neural circuits, the degree of hierarchy and plasticity between modules, and the possibility of forming new modules remain undefined design principles. As such, there are many unknowns regarding specific implementation, and research attempting to implement GWT is focusing on designs that combine GWT with other theories and technologies Franklin et al. (2013); Ito et al. (2023); Dossa et al. (2024); Huang et al. (2023); Garrido-Mercháin et al. (2022).

While the above GWT studies mainly focus on the question of “what information processing structures do we use,” there are attempts to examine GWT’s information processing from the question of “why did we arrive at this kind of information processing structure.” From the biological and evolutionary perspective, we can address this question by considering how such a structure might have provided adaptive advantages in terms of survival and reproduction Juliani et al. (2022). Examining the advantages of such information processing structures is an important theme that contributes to the construction of systems with advanced intelligence and flexible problem-solving abilities. In previous research, the focus has often been placed on the part of GWT’s information processing structure related to competing and integrating information among multiple specialized modules operating in parallel (Selection process) and on the part that shares the selected information with the entire system (Broadcast process), and the advantages and benefits of these have been discussed.

### 2.2 Functional advantages of selection

In this paper, the process of selecting information from among the information processed in parallel by multiple specialized modules and then integrating this information in a global workspace is called the “Selection” process.

#### 2.2.1 Diverse perspectives

By comparing and examining the outputs of multiple specialized modules, it is thought that it will be possible to generate a wider variety of solutions and ideas for a given task Ito et al. (2023); Wiggins (2012). For instance, if both a visual module and a language module are operating simultaneously, approaches that capture a problem from a pictorial/imaginative viewpoint can be compared with those that capture it from a linguistic/logical viewpoint. This concept is akin to the notion of “ensemble learning” Polikar (2012): by combining multiple models or modules with different specializations, the combination of models can complement the diverse aspects that a single model alone would not capture, thereby producing higher predictive accuracy and robustness overall.

Furthermore, the mechanism that integrates multiple parallel modules enables unexpected combinations of knowledge and skills from each module, which is thought to lead to creative thinking VanRullen and Kanai (2021); Wiggins (2012). For example, imagine a module responsible for visual thinking, inspired by metaphorical expressions provided by a language processing module, giving rise to a new diagram or prototype, which is then validated by a logical reasoning module. Alternatively, a module specializing in reinforcement learning might combine with a sensorimotor module’s proposed action strategy, leading to previously unanticipated solutions or task-execution procedures. The process of generating these incidental or divergent ideas and then evaluating, narrowing down, and integrating them is considered by many to be at the core of creative thinking Stojanov and Indurkhya (2013).

#### 2.2.2 Transfer learning

When faced with a new task, utilizing the skills already acquired in the specialized modules reduces the need to learn from scratch, and as a result, it is thought that the efficiency and speed of learning will improve VanRullen and Kanai (2021); Wiggins (2012). For instance, if there are modules that excel in visual recognition, language processing, or logical reasoning and each is independently trained, then when facing a new domain or a different task, it becomes possible to adapt quickly by making use of the knowledge and representations already accumulated in these modules. This is analogous to “transfer learning” Tan et al. (2018) in machine learning. In fact, when adapting a deep neural network learned in one domain (source domain) to another domain (target domain), reusing the lower-level feature extraction parts shortens the early training phase while still delivering high performance.

### 2.3 Functional advantages of broadcast

In this paper, the process of sharing selected information with all specialized modules is called the “Broadcast” process.

#### 2.3.1 Shared attention

It is thought that broadcasting allows each specialized module to concentrate its resources on information that is deemed to be extremely important according to the current goals and environmental conditions, thereby improving the efficiency and accuracy of task execution Wiggins (2012); Dossa et al. (2024). For example, consider a robot endowed with multiple sensory modules for vision, hearing, and touch, which is tasked with detecting, identifying, and accurately grasping an object. First, the visual module, operating unconsciously, generates multiple candidates, performing tasks such as location estimation and object classification in parallel. Meanwhile, the hearing module tries to gather hints from environmental sounds or voice commands that could modify actions. The tactile module prepares feedback control for the stage at which the robot actually grasps the object. After the information generated by each module is integrated by the Selection process, if the decision “to combine accurate location estimation from the visual module with minor corrective commands from auditory instructions” wins, that information is shared with all modules via the Broadcast function. As a result, the robot can carry out the plan “move the arm toward the coordinates estimated visually, corrected by auditory information” in coordination across all modules.

This mechanism seems to be highly relevant to the “Transformer architecture” Vaswani et al. (2017). Transformers, which demonstrate extremely high performance in various tasks such as natural language processing and image recognition, have a core mechanism known as “self-attention”. In self-attention, the inputs (or feature vectors) compute their mutual relevance, enabling the network as a whole to incorporate necessary contextual information. This mechanism is akin to GWT’s claim of handling diverse information while spotlighting important items and sharing them throughout the system. Though the transformer was not initially designed with the goal of mimicking consciousness, the fact that it achieves such high performance in language processing, image recognition, and more by way of sharing of important information hints at the fundamental usefulness of a strategy that shares the most crucial elements globally in an intelligent system.

#### 2.3.2 Predictive coding

Among the specialized modules, there are those that receive data from sensors (e.g., visual, auditory, tactile). If they receive predictions or metacognition as broadcast information, it may enhance the performance of the module’s output VanRullen and Kanai (2021); Wiggins (2012). For example, when the visual module is only processing lower-level features such as raw pixel data and edge information, it will only output tentative recognition results based on local statistics and pattern recognition. However, when higher-level context and objectives such as “this scene is outdoors and there is a high possibility that there are multiple people in the picture” and “the task is to judge the facial expressions of specific people” are broadcast from the global workspace, the visual module will re-evaluate its output while referring to these predictions and hypotheses. As a result, corrections such as prioritizing the extraction of resolution and regions of interest that are appropriate for the task, or more carefully searching for clues to separate people and backgrounds, can be expected to improve recognition performance and reduce false positives. This aligns closely with the concept of “predictive coding” Friston and Kiebel (2009) often discussed in neuroscience and cognitive science. Predictive coding posits that the brain or cognitive system is constantly sending top-down predictions from higher (i.e., more advanced) modules to lower (i.e., more basic) modules, while the lower-level modules calculate and return the discrepancy (prediction error) between the actual sensory input and the prediction. If the discrepancy is large, it implies that something different from the predictions is likely in the scene, and this error is returned upstream so that the higher-level module can update or generate new predictions. If the discrepancy is small, it implies that the prediction and actual data largely match, thus increasing the likelihood that it is really as observed. Through repeated mutual interplay between top-down predictions and bottom-up prediction errors, the entire perception and cognition system dynamically adapts to the environment.

### 2.4 Temporal dimension perspective in information processing

In this paper, information processing with the temporal dimension refers to a series of information processing operations that require careful consideration in order to find an answer, or that respond to a dynamically changing environment.

The former, a series of information processing, refers to focusing on the way functional processing is stacked. In many cases, an information processing operation can be decomposed into functional processing components. For example, an LLM is hierarchically structured with multiple functional processing components such as word tokenization, position embedding, weighting via self-attention mechanisms, and sequence generation, with each stage performing information transformation Vaswani et al. (2017). While we can describe an LLM as a single functional information processing system, from a temporal perspective, we will focus on the process of how functional processing steps that constitute an LLM are stacked. The focus on this approach of stacking functional processing has also gained attention in LLM research in recent years. Traditional LLM research has primarily focused on improving the performance of a single model by increasing the number of parameters or expanding the amount of training data, that is, an approach based on scaling laws, where model size is expanded to achieve more complex and advanced information processing capabilities Kaplan et al. (2020). However, in recent years, research has progressed toward designing the functional structure of processing in a more conscious manner by sequentially linking and integrating the outputs of multiple LLM models with distinct processing functions, rather than relying on a single large model for batch processing Sahoo et al. (2024). This study focuses on GWT and discusses what can be achieved through an infinite loop of selection and broadcast processing steps.

The latter refers to the ability to flexibly adjust internal processing strategies and outputs in response to a dynamically changing environment, where external conditions and demands are constantly changing. There are two main aspects to adapting to such dynamic environments. The first is the ability to adaptively change the next action selection or information processing policy based on the gradual updating of the representation of the internal state through continuous interaction with the environment Lesort et al. (2020); Shaheen et al. (2022). In this aspect, it is necessary to reconstruct the processing strategy in accordance with the current context, taking into account past experiences and the results of the most recent interaction. The second is the reactive response ability to immediately respond to sudden changes in the external environment and initiate appropriate processing or actions Shiwa et al. (2008); Alhaddad et al. (2020). This paper focuses not on the relationship between the required responsiveness and processing delays, but rather on how responsiveness is structurally enabled. Specifically, it examines what components and processing mechanisms must be combined to enable immediate responses to changes, and how these are realized through a division of roles within the overall system.

## 3 Hypothesis

In this paper, in addition to the structural advantages from each of the traditional GWT perspectives (Selection and Broadcast), we newly focus on the advantage of a cycle structure in which information processing occurs through Selection and Broadcast (Selection-Broadcast Cycle). Within this cycle structure, we discuss the dynamic, stepwise information processing in which Selection and Broadcast intertwine in parallel and intermittently. Note that this paper does not limit specialized modules and assumes them to be as broad as possible.

### 3.1 Dynamic thinking adaptation

The Selection-Broadcast Cycle possesses a structure that can realize any order of serial processing steps of specialized modules. The serial processing referred to here means processing that is carried out step by step (e.g., a chain of thought Wei et al. (2022), inductive and deductive reasoning Shanahan (2025). In contrast to parallel processing, in which multiple modules operate simultaneously, serial processing involves processing being carried out in order, with the information generated or selected by one module being passed on as input to the next module. In serial processing, the final answer is derived from the inferences and logical development that take place in the intermediate processing. This process of deriving conclusions in steps allows for reliable problem solving and decision making in various complex tasks while using only a small number of inferences and limited logical knowledge. For example, by simply memorizing the results of addition and multiplication of 0–9 and the methodology of longhand arithmetic, you can calculate any addition or multiplication of integers (e.g., 11 × 2 = 10 × 2 + 1 × 2). In this way, by breaking down complex tasks into simpler sub-tasks (i.e., tasks that can be processed using limited memory or simple rules) and dealing with them in stages, it is possible to deal with a wide range of different tasks using relatively little memory capacity.

**FIGURE 2:**
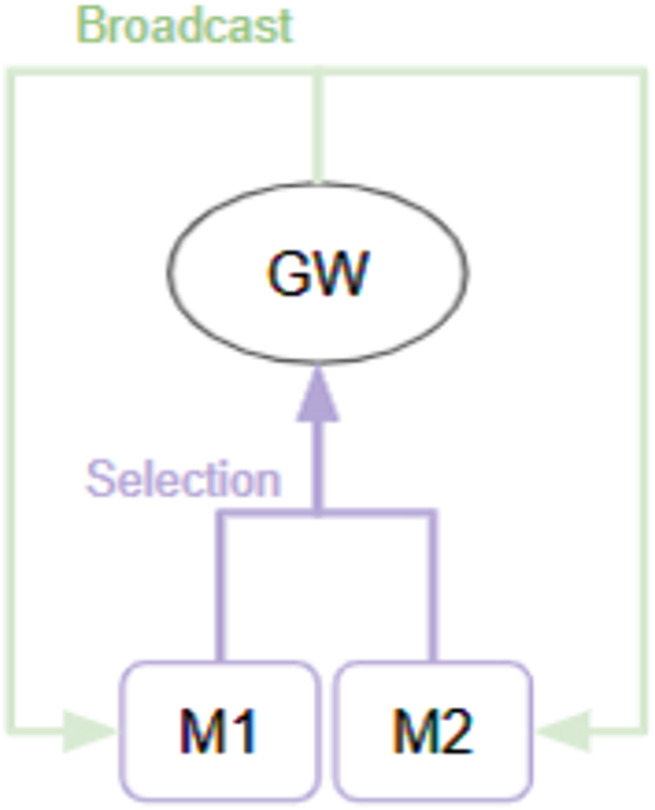
Example of GWT-based structure with two modules: This shows a GWT-based structure consisting of two modules (M1 and M2). Information output from the M1 and M2 module is selectively broadcast through the global workspace.

**FIGURE 3:**
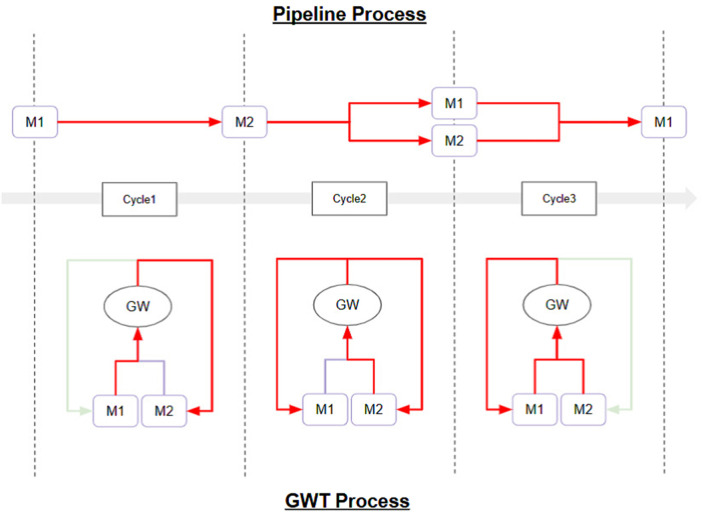
Conceptual Processing Flow of Dynamic Thinking Adaptation: This is conceptual processing flow based on the structure shown in Figure 2 for Dynamic Thinking Adaptation. The upper part shows an example of the module execution procedure (pipeline process), and the lower part shows the flow of processing to be executed in the Selection-Broadcast Cycle for the selected execution procedure. The red arrows indicate the flow of valid information. In Cycle1, the output from Module M1 is input into Module M2. In Cycle2, the output from Module M2 is input into both Module M1 and Module M2. In Cycle3, the outputs from both Module M1 and Module M2 are input into Module M1. In all cycles, the GWT-based structure enables information processing on the same structure by appropriately switching the selection.

The Selection-Broadcast Cycle process has a space where such intermediate inferences and logical developments can be freely performed. Figure 2 shows an example of a simple Selection-Broadcast Cycle structure with two modules (M1, M2). The upper part of Figure 3 shows conceptual processing flow of the execution procedure of modules, and the lower part shows the processing flow that executes that execution procedure in the Selection-Broadcast Cycle. As you can see, the Selection-Broadcast Cycle process can execute any execution procedure using the modules by switching the selection well. In order to implement such a vast serial processing space for intermediate inferences and logical development as a pipeline, a large tree structure made up of a large number of modules is necessary. The Selection-Broadcast Cycle process is thought to be a structure made up of a minimum number of modules using looped information processing.

Furthermore, this function enables flexible and dynamic processing, allowing the system to try out various thought processes and change your thought processes in response to changes in the situation. This is a great advantage when dealing with situations that are difficult to handle with a fixed pipeline process, such as when the processing procedure is unclear or the goal is changed partway through. For example, consider the case where a robot explores a room based on information from multiple sensors (vision, touch, audio input, etc.). At the start of the search, the main objective was to search for and move along the shortest route, and the processing was set up to call the object detection module and the route planning module in order. However, during the search, there were many collisions with people in the room along the route. In this case, the Selection-Broadcast Cycle makes it possible to share the problem with the whole system, devise a solution, and make changes to the processing, for example, by calling a human detection module while planning a route. Also, if a voice instruction is received and the content of the instruction changes, it is possible to call a voice recognition module to share the analysis results with the whole system, and then reconfigure the execution order of the visual module and route planning module in response to the results. Thanks to this variable serial processing, the order in which the necessary specialized modules are called can be flexibly rearranged in response to changes in the situation or new goals, making it possible to accomplish tasks that would be difficult with fixed pipeline processing.

This function also makes it possible to exchange information between any of the modules. VanRullen and Kanai (2021) point out that the global workspace functions as a “hub” between specialized modules, and that cycle-consistency learning Zhu et al. (2017) can be carried out by exchanging information between the same specialized modules. Cycle-consistency learning is a learning method that imposes constraints on the model to maintain consistency when converting data back and forth. These constraints ensure that once converted data can be restored to its original state by reversing the conversion, and prevent the loss of content or meaning during the conversion process. A major advantage is that it can learn domain mapping even without training data. In this way, the outputs of each specialized module are continuously cross-checked by repeating the Selection-Broadcast Cycle, and the entire system has the potential to detect potential inconsistencies, correct errors, and gradually build more reliable processing results.

Dynamic Thinking Adaptation holds the potential for systems to flexibly switch among multiple modes of thinking depending on the situation, thereby enabling adaptive problem solving and decision making. However, this approach presents several structural challenges. One significant issue is that implementing dynamic thinking adaptation requires a sophisticated selection mechanism to determine which module outputs should be elevated to conscious processing, and such a mechanism remains a major implementation hurdle. In particular, under dynamically changing module sets, maintaining an appropriate module selection strategy becomes a difficult task. Another issue is that the presence of dynamic thinking adaptation does not necessarily guarantee optimal solutions. The high degree of flexibility in strategy switching can lead to a risk wherein the system fails to maintain a consistent behavioral policy. There is also the possibility of processing delays caused by excessive contemplation. Thus, although dynamic thinking adaptation is a promising function, there remain substantial challenges in implementing and learning the selection control mechanisms that enable it.

### 3.2 Experience-based adaptation

As noted, in GWT, the information that is sequentially raised in the Global Workspace (Consciousness) through the Selection-Broadcast Cycle is shared with all specialized modules in a stepwise manner. Here, we focus on the point that the serial processing carried out in consciousness enters each specialized module in chronological order. It is thought that there are specialized modules that record such chronological consciousness and store it as experience memory Franklin et al. (2005). We can further suppose that such experience memory can be recalled if a similar situation arises. If so, it would become possible to speed up or predict the course of serial processing.

**FIGURE 4:**
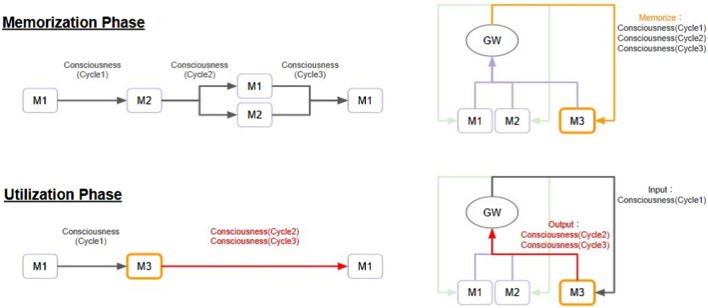
Conceptual Processing Flow of Experience-Based Adaptation: This figure shows a Selection-Broadcast Cycle structure consisting of two modules (M1 and M2) and one experience memory module (M3). The upper part shows the phase for storing information processing experiences (Memorization Phase), and the lower part shows the phase for outputting and utilizing the stored information processing experiences (Utilization Phase). In addition, the information processing flow is depicted in pipeline form on the left side and in GWT-based structure on the right side. In the memorization phase, information processing operations are assumed to be executed only in the M1 and M2 modules (pipeline on the upper top left). Consciousness (Cycle1), Consciousness (Cycle2), and Consciousness (Cycle3) refer to the information (i.e., global workspace state) sent in each respective cycle. Although the memorization phase consists solely of information processing operations performed by the M1 and M2 modules, consciousness information also reaches the M3 module, so Consciousness (Cycle1), Consciousness (Cycle2), and Consciousness (Cycle3) are sequentially stored in the M3 module (orange arrow in the GWT-based structure on the upper right). In the utilization phase, similar to cycle one of the memorization phase, when Consciousness (Cycle1) is broadcast, the M3 module outputs Consciousness (Cycle2) and Consciousness (Cycle3) as retrieved memories. This reaches Consciousness (Cycle3) one cycle earlier than in the memorization phase, enabling faster thinking, prediction of outcomes, or application of knowledge.

Figure 4 shows conceptual processing flow of a Selection-Broadcast Cycle structure with two modules (M1, M2) and one experience memory module (M3). Consciousness (Cycle1), Consciousness (Cycle2), and Consciousness (Cycle3) refer to the information (i.e., global workspace state) sent in each respective cycle. Consciousness (Cycle1), Consciousness (Cycle2), and Consciousness (Cycle3) are each listed in the global workspace in chronological order. Because these consciousnesses are broadcast in chronological order, they flow into the experience memory module as well. The experience memory module retains them as experiences. Then, when Consciousness (Cycle1) is broadcast again, the experience memory module can output Consciousness (Cycle2) and Consciousness (Cycle3) as recalled memories. This means that it is possible to reach the output of Consciousness (Cycle3) in two cycles, whereas it would have taken three cycles to reach it in the past. As described above, it is possible that the Selection-Broadcast Cycle will enable faster serial processing and prediction. This is similar to the concept of “chunking” Gobet et al. (2001) in cognitive science, and if learned schemas and procedures are stored as a kind of “chunk”, then when faced with a similar task next time, that chunk can be called up all at once to quickly progress with the processing.

This mechanism not only increases processing speed, but also promotes inference and anticipation of actions. In other words, while referring to past thought processes, it is possible to make predictions such as “there is a possibility that new information will be lacking at this stage” or “it would be better to activate the sensorimotor module before the logical inference module in the next step”, and it is possible to adjust the order of module calls and resource allocation in advance based on these predictions. As a result, each step in the variable serial processing is no longer a simple “trial and error” process, but rather a planned and efficient process that makes full use of past accumulated knowledge. The meta-cognitive decisions made during this process, such as “which module should be activated at what time” and “when should top-down information be updated”, are also optimized through the use of overall information sharing and memory via the Selection-Broadcast Cycle. In this way, by having a system in place that can record and utilize a record of serial processing, it is hoped that the cognitive architecture based on GWT will not only speed up, but also acquire advanced problem-solving capabilities that incorporate reasoning and prediction with an eye on the next move.

There have been several implementations of agent systems that apply experience memory as knowledge (e.g., reasoning and prediction) Laird et al. (2012); Martin et al. (2021). For instance, Franklin and colleagues Franklin et al. (2013) have demonstrated a framework called LIDA (Learning Intelligent Distribution Agent), which builds on GWT to incorporate conscious content into various cognitive modules, including an episodic memory module. In LIDA-based implementations, information that reaches consciousness is not only broadcast to specialized modules but is also chronologically recorded in episodic (or experience) memory. When a similar situation occurs, the system recalls the sequence of recorded conscious events and applies them as learned knowledge.

Experience-Based Adaptation is a framework in which a system incrementally improves its behavior according to the situation by accumulating and referencing past episodes or historical information. Nevertheless, this approach faces challenges primarily in experience management and generalization in learning. First, there exists a trade-off between memory capacity and retrieval efficiency. As more experiences are accumulated, the number of episodes that must be referenced increases, leading to greater search time and complexity in memory management. In applications requiring fast inference, such delays can undermine practical usability. Second, in domains where the interpretation of experience is highly context-dependent, erroneous generalization becomes a critical issue. Strategies that were successful in the past may often fail in slightly different contexts, and distinguishing between applicable and inapplicable past knowledge requires a high degree of abstraction ability.

### 3.3 Immediate real-time adaptation

**FIGURE 5:**
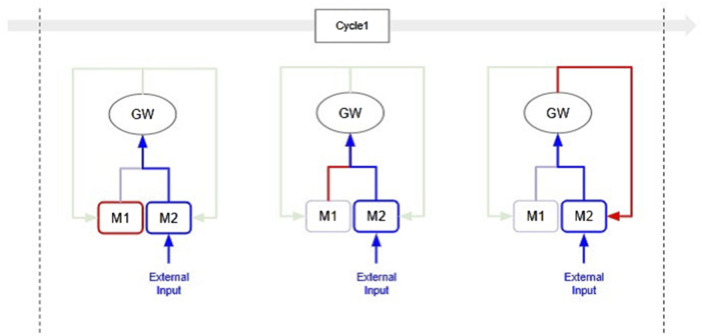
Conceptual Processing Flow of Immediate Real-Time Adaptation: This is conceptual processing flow for Immediate Real-Time Adaptation based on the Cycle1 shown in Figure 3. The process of Cycle1 is broken down into the execution process of the M1 module (left), the selection process of the output results of the M1 module (center), and the broadcasting process of the output results of the M1 module (right), with red lines highlighting these processes. The blue lines indicate the path taken by external inputs as they are raised to the global workspace through the processing of the M2 module. In the left figure, external input intervenes during the execution of the M1 module. In the center figure, external input is recognized simultaneously with the M1 module. In the left figure, external input intervenes while the output results of the M1 module are being broadcast. The key point here is that external input can intervene at any stage of the Selection-Broadcast Cycle.

The Selection-Broadcast Cycle enables real-time intervention in the results of intermediate processing based on external input. Figure 5 shows a simple scenario in which external intervention occurs in a Selection-Broadcast Cycle process consisting of two modules (M1 and M2). As shown in the figure, external inputs can affect the serial processing of the global workspace at any point. For example, when a specialized module detects external important information, this information can be immediately input into the global workspace through the Selection process and then distributed to all other modules via the Broadcast process. It is important to note that this immediate input is not necessarily blocked because other modules are executing. This is because the modules operate in parallel and asynchronously. In general pipeline processing, if a module is executing, all processing of any input is held until that execution is complete. In the Selection-Broadcast Cycle, this fast path significantly reduces unnecessary waiting time and message transmission time, greatly improving the responsiveness of real-time systems.

In practical robotics scenarios, such flexible intervention mechanisms have notable advantages. For instance, imagine a robot performing an assembly task using multiple sensory modules (visual, tactile, auditory). Suppose the robot’s tactile sensor suddenly detects an unexpected slip or instability in its grip. With the Selection-Broadcast Cycle, this critical information is rapidly promoted into the global workspace, interrupting the ongoing processing sequence. Consequently, other modules (e.g., motor control, vision processing, or reinforcement learning) immediately receive this alert and can swiftly initiate corrective actions. This immediate broadcast enables the system to promptly reconsider and revise its gripping strategy from both top-down (strategic re-planning) and bottom-up (sensor-driven adjustments) perspectives, substantially improving safety, precision, and robustness in real-time.

Immediate Real-Time Adaptation holds the potential for systems to instantly respond to environmental changes by determining and modifying behavioral policies on the fly. However, this mode of adaptation raises the issue of how to maintain internal state consistency and processing stability when new information interrupts ongoing processes. Specifically, systems must be designed to interrupt and reassess ongoing module processing in the presence of newly incoming high-priority information, while also managing priorities to avoid overreacting to low-relevance perturbations. In environments with frequent external inputs, repeated interruption and resumption may increase overhead and task-switching burdens, potentially leading to decreased overall efficiency. While immediate adaptation is inherently useful, excessive reliance on it can deprive the system of the opportunity to engage in sustained inference, thereby posing a risk of unstable performance.

## 4 Discussion

### 4.1 Extending GWT to the temporal dimension

Traditional discussions of GWT’s intelligence have predominantly emphasized the process on static, supervised settings, which rely heavily on pre-labeled data sets, explicit instructions, and predefined tasks (e.g., ensemble learning, transfer learning, self-attention, predictive coding). In such scenarios, intelligence manifests primarily as a system’s ability to accurately replicate patterns and knowledge derived from historical, structured data. However, the real-world application of artificial intelligence increasingly demands a shift toward dynamic, unsupervised settings, where tasks, environments, and goals continuously evolve, often without explicit guidance or labeled examples.

In dynamic, unsupervised scenarios, intelligent systems face fundamentally different challenges. Rather than relying on historical labels or fixed benchmarks, these systems must autonomously discover meaningful patterns, adapt swiftly to changing contexts, and continuously learn from ongoing experiences. In this paper, we discussed the strengths of GWT in such real-time processing by focusing on Selection-Broadcast Cycle. We explained that this Selection-Broadcast Cycle realizes flexible processing, is capable of being accelerated, and is a mechanism that can respond immediately to real-time changes. Thus, by highlighting the advantages of the Selection-Broadcast Cycle, this paper extends traditional conceptions of GWT intelligence into the realm of dynamic, unsupervised learning, opening new pathways toward the development of more robust, adaptive, and autonomous artificial intelligence systems capable of thriving in complex real-time environments. Future research could further explore practical implementations and empirical evaluations to validate these theoretical insights and expand the applicability of GWT-based architectures in diverse, real-world scenarios.

Furthermore, although GWT seems well-suited for thriving in the real-time world, one potential way to enhance its adaptability further could involve multiple consciousness (GWT) processes operating in parallel. This parallelization could facilitate the simultaneous exploration of diverse solutions, enhance adaptability by rapidly responding to varied and unpredictable changes, and effectively distribute cognitive load, thereby potentially surpassing the limitations inherent to a single, centralized consciousness structure. Such a mechanism might represent the collective intelligence observed in groups of humans, suggesting that human societies themselves could represent natural exemplars of parallel consciousness networks capable of robust, adaptive decision-making in complex and dynamic environments. For example, Taniguchi (2024) is researching the dynamics of such group intelligence and language development.

### 4.2 Limitations and future work

While the proposed Selection-Broadcast Cycle structure inspired by the Global Workspace Theory (GWT) provides a compelling theoretical framework for adaptive, real-time cognitive architectures, several critical limitations need to be acknowledged and addressed in future work.

One significant limitation of this study is the absence of empirical validation. The advantages of the Selection-Broadcast Cycle, such as dynamic thinking, experience-based acceleration, and immediate real-time responsiveness, remain largely theoretical. Currently, the paper does not present experimental results, simulations, or quantitative analyses to substantiate these claims. Therefore, readers must accept the described benefits without direct evidence of improved adaptability or efficiency compared to other existing methods. To strengthen future iterations of this research, practical implementations such as comparative simulations or robot-based experiments demonstrating fewer task failures or quicker adaptation would be essential.

For example, these adaptations share common challenges related to scalability and costs. First, there is the issue of expanding computational resources. In Dynamic Thinking Adaptation, the order space of sequential processing grows exponentially, leading to a sharp increase in the cost of maintaining optimal module selection. In Experience-Based Adaptation, the contents of consciousness at each cycle are continuously accumulated as experiential memory, which tends to cause memory bloat and delays in retrieval processes. In Immediate Real-Time Adaptation, frequent interruptions by external inputs increase overhead and task-switching costs, resulting in a trade-off between real-time responsiveness and overall efficiency. All of these problems become more severe as the number of modules and the complexity of tasks increase. Second, there is the burden of information management. In Dynamic Thinking Adaptation, the exploration of complex sequential patterns can cause unnecessary processing or the accumulation of redundant information. In Experience-Based Adaptation, neglecting the abstraction or compression of stored memories leads to “memory overload,” which can hinder generalization capability. In contrast, Immediate Real-Time Adaptation faces a heightened risk of internal state inconsistency due to frequent external interruptions.

Another point is that the proposed selection and broadcast cycle structure has not been sufficiently compared with existing information processing architectures. In recent years, various architectures with different theoretical foundations have been proposed, such as Recurrent Processing Theory, Computational Higher-Order Theories, Attention Schema Theory, and Information Generation Theory Butlin et al. (2023); Juliani et al. (2022). These theories partially realize information selection, attention allocation, and integration of memory and control in different forms. By comparing these methods with the selection-broadcast cycle proposed in this study, the structural characteristics, advantages, and limitations of the proposed approach can be clarified for “multimodal” and “parallel” architectures that execute tasks by simultaneously utilizing multiple cognitive functions. In addition, future work must also define concrete performance indicators to evaluate each of the three proposed adaptive mechanisms in controlled and comparative settings.

## 5 Conclusion

In this paper, we explored the potential of the Global Workspace Theory (GWT) and, in particular, the Selection-Broadcast Cycle, as an information processing architecture suitable for dynamic, unsupervised real-time environments. Traditional approaches to artificial intelligence often rely heavily on structured, labeled data, where intelligence primarily involves replicating known patterns. However, real-world applications require systems that can continuously adapt and respond to evolving tasks, environments, and goals. In this context, we highlighted the Selection-Broadcast Cycle’s strengths: its flexibility to rearrange module execution order dynamically, prediction and acceleration capabilities based on experience, and its responsiveness to immediate real-time inputs.

Our hypothesis suggests that a cognitive architecture based on GWT and, specifically, the Selection-Broadcast Cycle, provides a robust framework for dynamic decision-making and rapid adaptation in complex environments. The ability to dynamically rearrange processing sequences, utilize experience-based memory, and respond quickly to changing conditions demonstrates the potential of GWT-based architectures to effectively address the challenges faced by real-time intelligence.

The practical feasibility of implementing robust and adaptive Selection mechanisms in real-world systems remains a critical unresolved question. Future research must address this challenge, potentially through integrating machine learning techniques and advanced evaluative frameworks, to further validate and extend the applicability of GWT-based architectures. By tackling these challenges, we can move closer to developing truly autonomous, flexible artificial intelligence systems capable of thriving in the complexities and uncertainties of the real-time world.

## Data Availability

The original contributions presented in the study are included in the article/supplementary material, further inquiries can be directed to the corresponding author.
